# Supplementary material for: Structure and mechanism of a Type III CRISPR defence DNA nuclease activated by cyclic oligoadenylate
Source: Nat Commun. 2020 Jan 24;11:500. doi: 10.1038/s41467-019-14222-x (PMC6981274; doi:10.1038/s41467-019-14222-x)
Supplement: Supplementary file 1 — Supplementary Information [file 41467_2019_14222_MOESM1_ESM.pdf]

Supplementary Information

Structure and mechanism of a Type III CRISPR defence DNA nuclease activated by cyclic oligoadenylate

Stephen A McMahon[1,3], Wenlong Zhu[1,3], Shirley Graham[1], Robert Rambo[2], Malcolm F White[1]*, Tracey M Gloster[1]*

Supplementary Figures

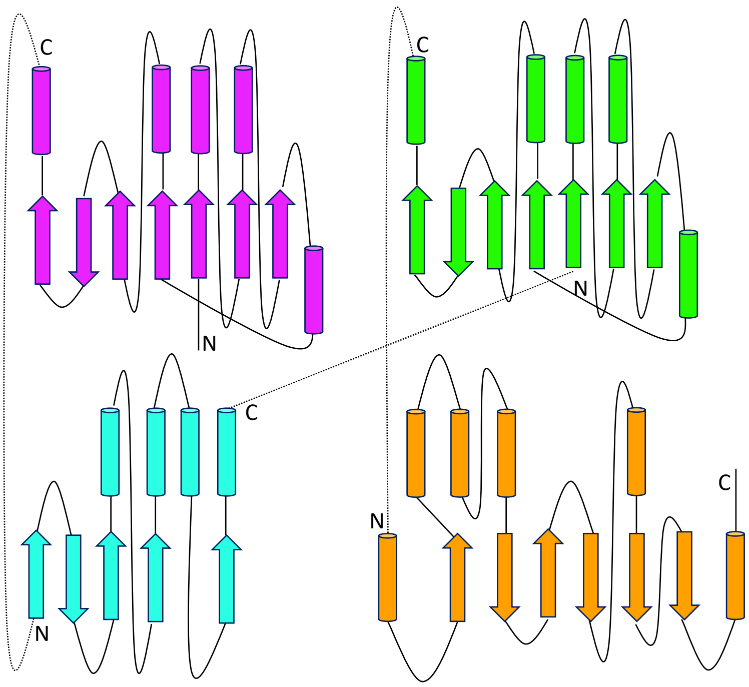

Supplementary Figure 1. Schematic of Can1 topology. α-helices are represented by cylinders and β-sheets by arrows (which indicate the direction from N- to C-terminus). The first CARF domain is shown in magenta, nuclease-like domain in cyan, second CARF domain in green, and nuclease domain in orange. The N- and C-terminus for each domain is indicated. Loops between domains are shown as dashed lines.

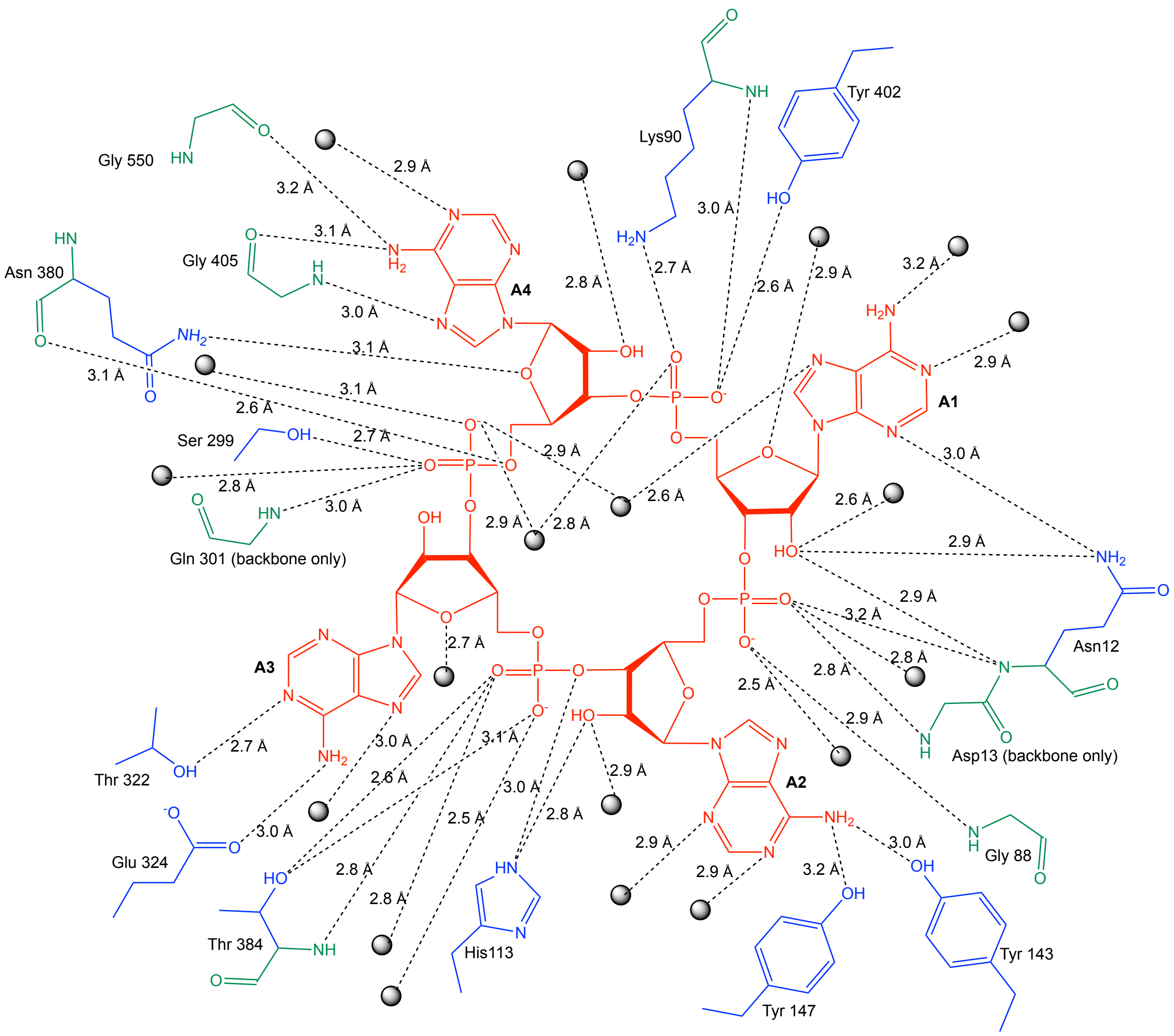

Supplementary Figure 2. Schematic showing interactions between Can1 and cA_4. cA_4 is shown in red, interactions with main chain atoms in green, interactions with side chain atoms in blue, and water molecules are represented as spheres. AMP moieties are numbered A1-4. Hydrogen bonds are represented as dotted lines, annotated with the distance.

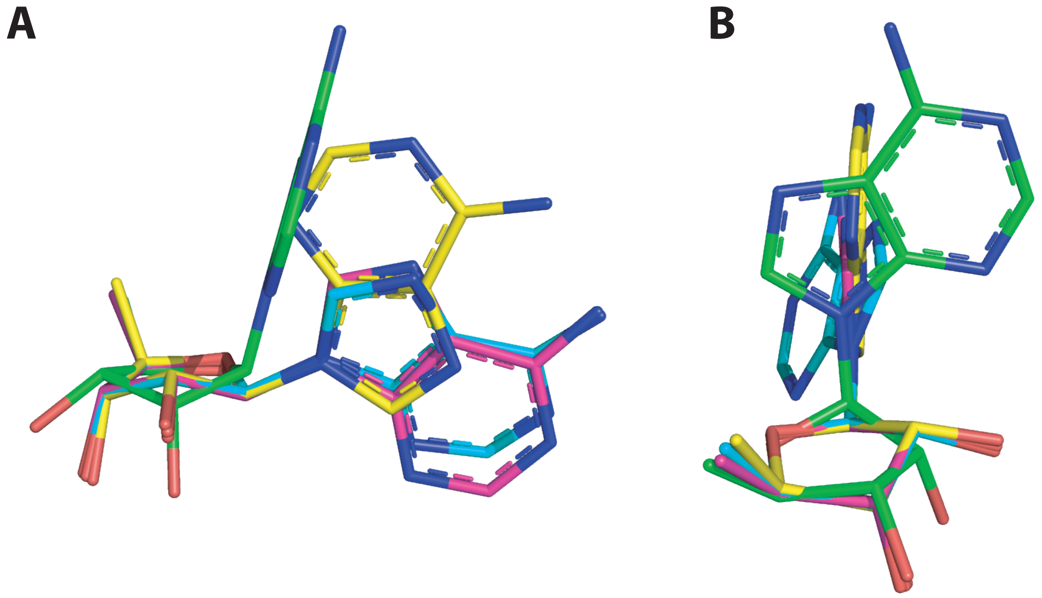

Supplementary Figure 3. Superposition of the four adenosine units in cA_4. The superposition is shown from two different views (**A** and **B**), with each adenosine unit indicated by a different colour. There is a marked difference in the conformation of the ribose ring and position of the adenine base for one of the units (shown in green) which is brought about by a π-π stacking interaction with Trp42.

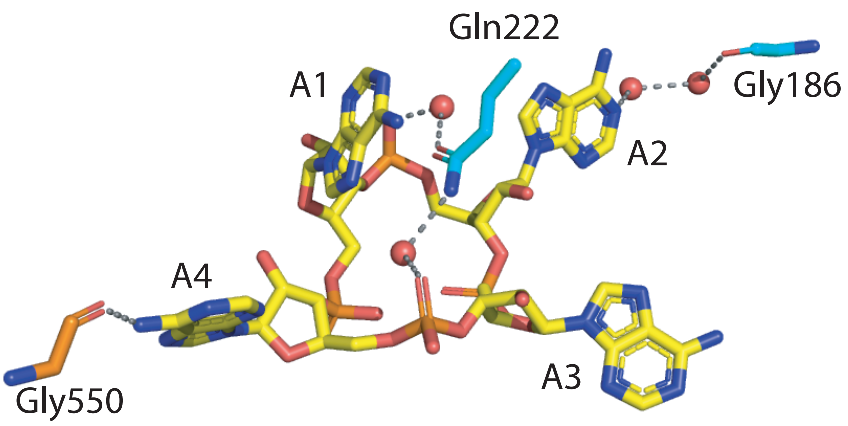

Supplementary Figure 4. Interactions of non-CARF domain residues with cA_4. cA_4 and residues are shown in stick representation, with cA_4 in yellow and amino acid residues coloured by the domain from which they originate (domain 2: cyan; nuclease domain: orange). The dotted lines represent hydrogen bond interactions, and red spheres represent water molecules.

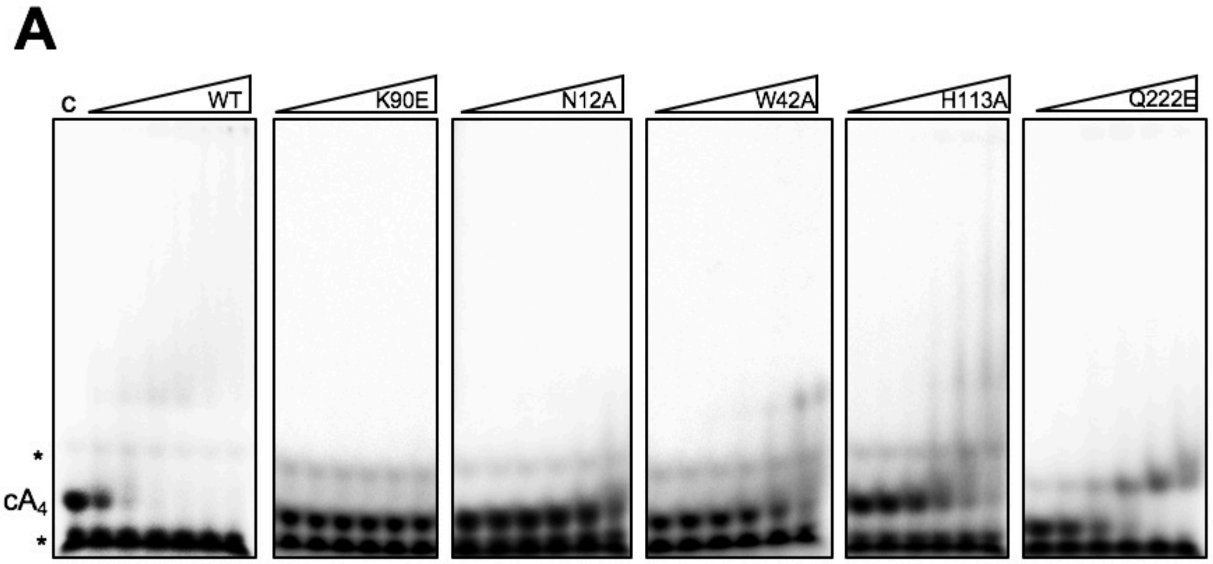

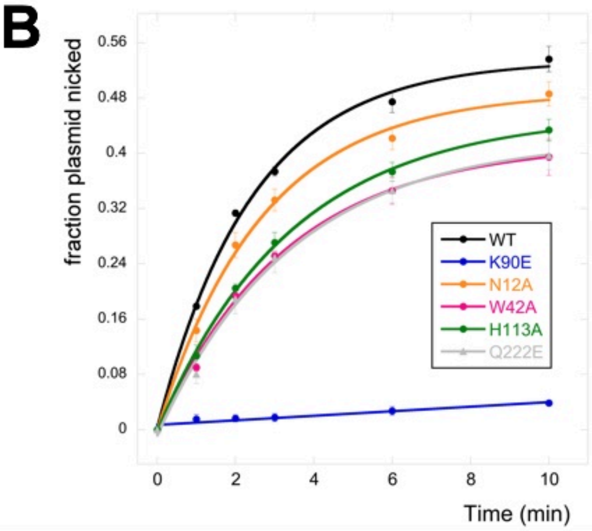

C

Variant	App Kd (µM)	Activity (min^{-1})
WT	0.1	0.41 ± 0.03
K90E	>>4	0.003
N12A	4	0.37 ± 0.03
W42A	2	0.31 ± 0.03
H113A	1	0.29 ± 0.01
Q222E	0.5	0.29 ± 0.04

Supplementary Figure 5. Investigation of cA4 binding residues. (**A**) Electrophoretic mobility shift assay of ^{32}P labelled cA_4 (100nM) incubated with Can1 and variants (K90E, N12A, W42A, H113A and Q222E) for 10 min at room temperature in the presence of 100 mM NaCl, 1 mM EDTA, 5 mM $MnCl_2$ and 2 µM BSA. Lane c indicates the reaction incubated without protein. The protein concentrations used in this assay are 0.1, 0.25, 0.5, 1, 2 and 4 µM. ^{32}P labelled cA_4 was prepared by incubating ^{32}P-α-ATP with cyclase. * indicates side products of the cyclase activity. (**B**) Kinetic analysis of supercoiled plasmid nicking by Can1 and variants. Experiments were carried out by incubating Can1 and variants with plasmid pEV5HisTEV under the conditions described in Figure 4. To differentiate nuclease activity of wild type Can1 and all variants, the kinetic assay was changed by reducing protein and cA_4 concentration to 100 nM and 20 nM, respectively. Data points are the mean of triplicate experiments with the standard deviation shown. (**C**) Approximate K_d and activity rate constants of Can1 and variants (K90E, N12A, W42A, H113A and Q222E) from the assays. Source data are provided as a 'Source Data' file.

```
Can1-N    1       MQAPVYLCLLGNDPAPAYLG-LKVVEREAGRVAKAVFYSFPAWNEEYGKKRQAFFR  55
Can1-C  285 LPLPQEGPLLLALVSEQAVPLYAAYLHAGPR--------EVYLLTTPEMESRLRWA--EAF 335
VC1899    1         MAIHVGIIDQDPVRLVTPLLDHRTV-----SRHIIFIGDHTQTVIYQRLSDVLN  49

Can1-N   56 LLSEKGVLYEERP-----------LEKGLEEAEAR-----EVWVNLTGGAKYWAVRFLGH  99
Can1-C  336 FRGKGV--RVHRSFLSGPWALREVRDLLAPVVEEALRRGHPVHANLNSGTTAMALGLYLA 393
VC1899   50 KRNI----STDFFEIPAGSNTSAIKSAIRELAETLKARGEEVKFNASCGLRHRLLSAYEV 105

Can1-N  100 WRRPGARVFLVEGHRALEAPRALFLWPREEERSLEAEALT----LEEYARLYLEP----- 150
Can1-C  394 LR-DGARAHYLD------GDRLLLLDGGEAEVPWEEGRPEDLLALRGYRFEEEYPDARPD 446
VC1899  106 FRSYHWPIFVVE------PNSDCLCWLYPEGNNDTQVQD--RITIADYLTIFGARGEFNE 157

Can1-N  151 ---LGEAWERVSPPG--------------------------------------------- 162
Can1-C  447 PGLLALAEEILRRWDEVQTSW--------------------------------------- 467
VC1899  158 HQLSPQLDQQLYQLGERWASNALELGPGLATLNYLATTCRKEQKLDVELSDKQQGYRELN 217

Can1-N  163 ----------------------------------------------------------AF 164
Can1-C  468 -EASPLVRRFLKFWKKRFGQAFPPKRLSRLKGLPLEYAVYSHLN--AHLAPKGGQARMGG 524
VC1899  218 LLLSDLVEAKIASYENGILTFINEEARRFANGEWLETLVHSTVKQIQDDMPTIQDRSLNV 277

Can1-N  165 PPGAQAAR--LPGREGGVFVVHR--GLPYWYWVRPHLGGEAKDMSRKALSAFSGEAKRLG 220
Can1-C  525 HLVPLGGNEALAPQSTEVDGVFFHRGALWFVECKP-----TDEGLRERAPIMAELVRSVG 579
VC1899  278 QVYRQLGEREVRN---ELDVATVVNNKLHIIECKT--KGMRDDG-DDTLYKLESLRDLLG 331

Can1-N  221 GQLCLPVVPYHKAHLRSRHPKERENVFARWRAWAREYGVFLVDPGRP--------LEEEV 272
Can1-C  580 GVEARGLMVARR---------------WRGAPPPASPNLVYMALEGGEGVGVYRFPEELE 624
VC1899  332 GLQARAMLVSFR------------PLRHNDITRAEDLGLALIGP------DELKDLKTHL 373

Can1-N  273 ASLIKGKASKKA 284
Can1-C  625 KALSRNPAPRRG 352
VC1899  374 TQWFKAAGGN   383
```

Supplementary Figure 6. Sequence alignment. Sequence alignment based on structural homology of the first CARF domain and the nuclease-like domain of Can1 (Can1-N), the second CARF domain and nuclease domain (Can1-C) and VC1899. α-helices are highlighted in yellow and β-strands in cyan. Residues in Can1 that interact with cA_4 are coloured red, and predicted cA_4 interacting residues in VC1899 are coloured orange. Nuclease active site residues are coloured purple.

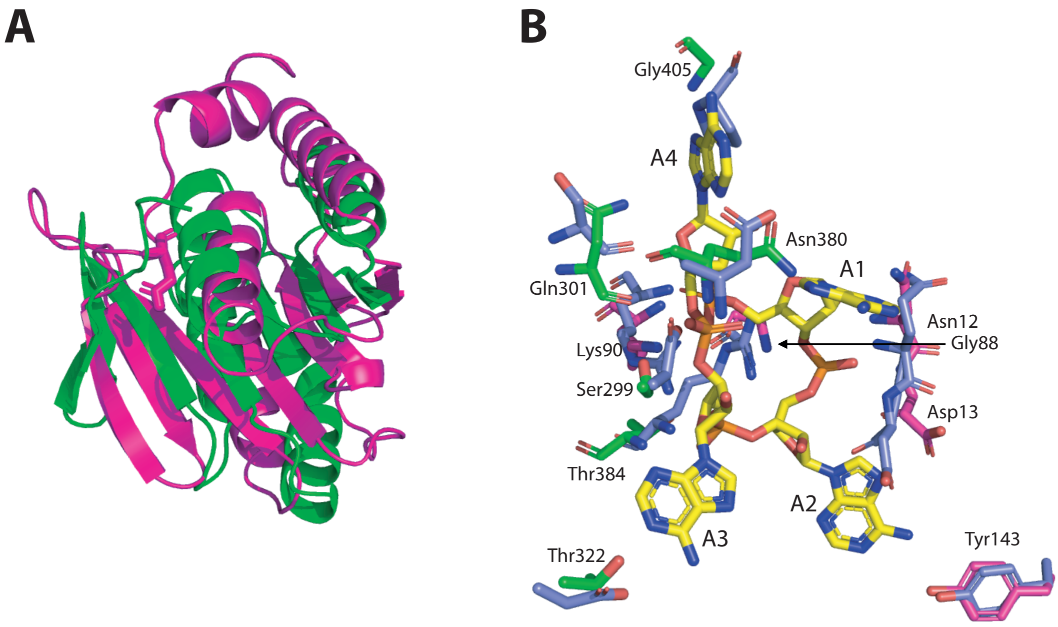

Supplementary Figure 7. Superimpositions of the CARF domains from Can1. (**A**) Superimposition of the two CARF domains of Can1. The first CARF domain is shown in magenta and the second CARF domain in green. (**B**) The binding site of cA_4 in complex with Can1, overlapped with VC1899. cA_4 and binding site residues are shown in stick representation, with cA_4 in yellow and amino acid residues coloured by the domain from which they originate (CARF domain 1: magenta, CARF domain 2: green). VC1899 (mauve) is superimposed onto the CARF domain of each 'half' of Can1, and those residues that are structurally conserved are shown. Numbering in the figure refers to residues from Can1. The equivalent residues in VC1899 are: Arg96 to Lys90 (CARF1) and Thr384 (CARF2), Gln10 to Asn12, Asp11 to Asp13 (CARF1) and Gln301 (CARF2), Gly94 to Gly88, Pro118 to Gly405, and Ser92 to Asn380 (all main chain interactions), and Arg96 to Lys90 (CARF1) and Thr384 (CARF2), Gln10 to Asn12, Tyr145 to Tyr143, Asp34 to Thr322, Asp9 to Ser 299, and Ser 92 to Asn380 (all side chain interactions).

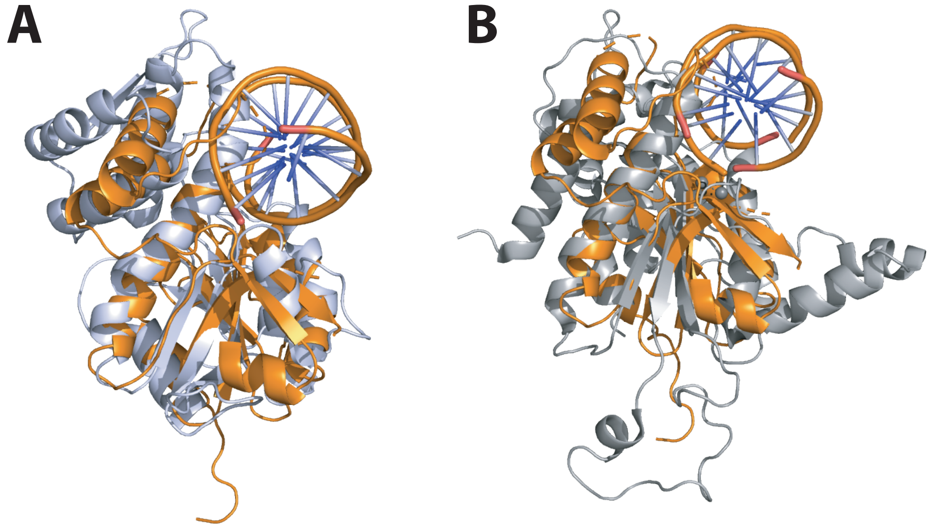

Supplementary Figure 8. DNA binding models for Can1. The nuclease domain of Can1 (orange) overlapped with (**A**) the nuclease domain of *Age*I (PDB: 5DWB) (light mauve) in complex with dsDNA shown in cartoon form and (**B**) the nuclease domain of *Ngo*MIV (PDB: 1FIU (light grey) in complex with dsDNA.

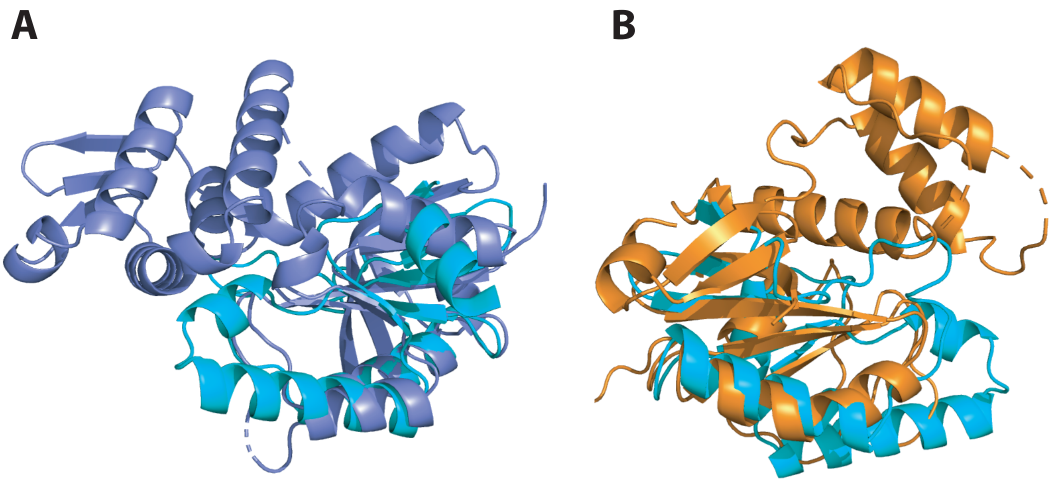

Supplementary Figure 9. Superimposition of the nuclease and nuclease-like domains of Can1. Superimposition of the nuclease-like domain of Can1 (cyan) with the nuclease domain of (A) VC1899 (mauve) and (B) Can1 (orange). The nuclease-like domain superimposes with around half of the secondary structure elements found in the nuclease domain. It lacks the N-terminal α-helix, β-strand, three α-helices (in a helix-loop-helix-loop-helix arrangement) and β-strand. The nuclease-like domain overlaps well with the next two β-strands, α-helix, and β-strand, and then has an insertion (with respect to the nuclease) of a helix-turn-helix motif followed by a β-strand. The final α-helix of both domains superimposes well.

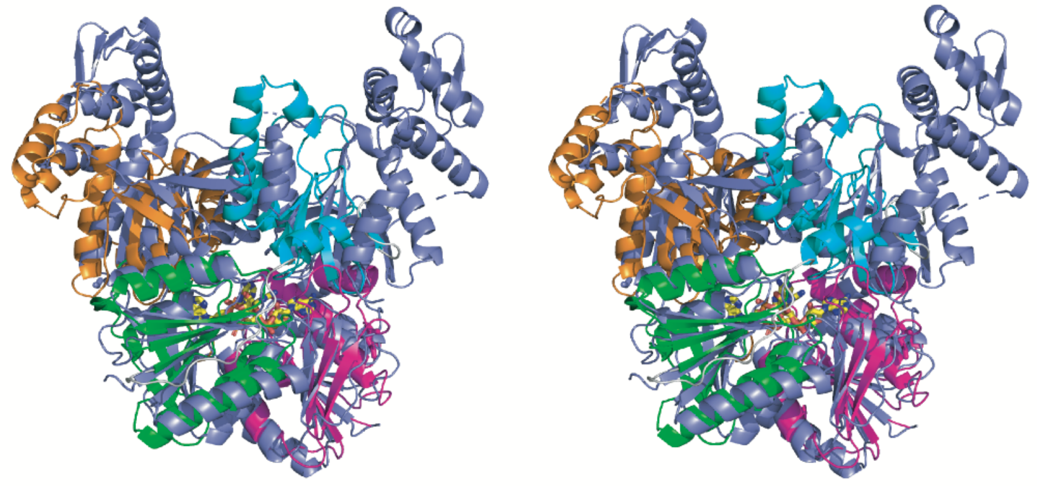

Supplementary Figure 10. Superposition of Can1 and VC1899. Divergent stereo representation of Can1 (first CARF domain, magenta; nuclease-like domain, cyan; second CARF domain, green; nuclease domain, orange; loops between domains, grey) with VC1899 (mauve). The superposition was performed on the CARF domains from each protein only, and thus reflects potential conformational changes in the open (as predicted by the structure of VC1899) and closed (Can1 in complex with cA_4) states of the nuclease and nuclease-like domains.

Supplementary Table 1. Synthetic gene encoding *can1*.

Two gBlock (IDT) codon optimised DNA fragments were used to incorporate the full gene into the vector. Bases shown in bold are the restriction enzyme sites and those in italics are overhangs.

Fragment 1, containing *Nco*I and *Bam*HI restriction enzyme sites.

*GGCG***CCATGG**AGGCGCCCGTTTATTTGTGCCTTCTTGGGAACGATCCTGCCCCCGCCTATCT
TGGTCTTAAAGTAGTAGAGCGTGAAGCTGGGCGCGTAGCTAAGGCTGTGTTCTATTCGTTCC
CAGCATGGAACGAGGAGTACGGCAAAAAACGCCAAGCGTTTTTTCGCCTGTTATCGGAAAAG
GGGGTCCTGTACGAAGAGCGTCCGTTAGAAAAAGGCTTGGAAGAGGCTGAGGCTCGTGAAG
TTTGGGTCAATTTAACTGGAGGAGCAAAATACTGGGCGGTACGTTTCTTGGGACACTGGCGT
CGTCCCGGGGCACGTGTTTTTTTAGTTGAAGGACATCGTGCCCTTGAGGCCCCTCGCGCCCT
GTTCTTATGGCCTCGCGAGGAAGAGCGCAGCCTTGAAGCGGAGGCCCTTACCTTGGAAGAAT
ATGCCCGTCTGTACCTGGAGCCCCTTGGCGAAGCATGGGAGCGCGTCAGCCCGCCAGGAGC
ATTTCCGCCGGGAGCGCAAGCTGCACGCTTACCAGGCCGTGAAGGCGGAGTCTTCGTAGTT
CATCGCGGCCTTCCTTACTGGTATTGGGTACGTCCCCACTTGGGAGGTGAGGCCAAAGATAT
GTCACGTAAGGCTTTAAGTGCATTTTCCGGTGAAGCTAAACGCTTAGGGGGTCAGTTGTGCTT
GCCCGTGGTTCCATATCATAAAGCTCACTTACGCAGTCGTCACCCGAAAGAGCGTGAGAATG
TATTCGCACGCTGGCGTGCCTGGGCTCGTGAATACGGGGTGTTCTTGGT**GGATCC**GGGT

Fragment 2, containing *Bam*HI, *Hind*III and *Xho*I restriction enzyme sites

TTCTTGGT**GGATCC**GGGTCGCCCTCTGGAAGAAGAAGTCGCCTCATTGATTAAAGGGAAGGC
TAGCAAGAAAGCCTTGCCATTACCTCAAGAAGGACCATTGTTATTAGCATTGGTCTCAGAACA
GGCAGTTCCTCTGTATGCGGCTTACCTTCACGCTGGCCCGCGTGAAGTTTACTTACTTACAAC
ACCAGAGATGGAAAGCCGTCTTCGCTGGGCAGAAGCCTTTTTTCGCGGAAAAGGAGTCCGTG
TCCACCGTAGTTTCTTATCCGGTCCGTGGGCGTTGCGTGAGGTGCGCGACTTGCTGGCGCCC
GTCGTTGAGGAGGCTTTGCGCCGTGGGCATCCCGTTCATGCGAATTTAAATTCCGGAACCAC
TGCTATGGCATTGGGGCTTTATCTTGCTCTTCGTGATGGGGCGCGCGCACATTACTTGGACG
GAGATCGTTTATTATTATTAGATGGCGGGGAGGCCGAAGTGCCCTGGGAAGAGGGCCGTCCC
GAGGACTTGCTGGCTTTACGCGGATACCGTTTTGAAGAAGAGTATCCGGATGCCCGTCCTGA
TCCCGGGCTGTTAGCGTTGGCGGAGGAAATTTTGCGTCGCTGGGACGAAGTGCAGACATCAT
GGGAAGCGAGTCCTCTTGTTCGTCGTTTTTTGAAGTTCTGGAAGAAGCGCTTCGGGCAAGCG
TTCCCACCAAAGCGCTTATCCCGTCTTAAAGGGTTGCCCTTAGAGTACGCAGTATATTCGCAC
CTGAATGCACATCTGGCCCCCAAGGGAGGCCAAGCTCGCATGGGAGGACATCTTGTCCCTCT
TGGAGGTAATGAGGCATTAGCCCCGCAATCCACTGAGGTCGACGGGGTATTTTTTCACCGTG
GCGCCCTTTGGTTCGTCGAGTGTAAACCAACCGACGAGGGATTACGTGAACGCGCCCCGATT
ATGGCTGAACTTGTTCGTAGCGTAGGAGGAGTCGAGGCACGTGGTTTAATGGTGGCGCGTCG
TTGGCGCGGGGCTCCTCCCCCTGCCTCCCCTAATCTTGTTTACATGGCATTGGAGGGAGGAG
AAGGGGTAGGTGTGTATCGCTTTCCGGAAGAATTAGAAAAGGCATTGTCGCGTAACCCGGCA
CCTCGTCGCGGG**CTCGAG**TGA**AAGCTT***GCGC*

Supplementary Table 2. Oligonucleotide sequences used for the site-directed mutagenesis of the gene encoding *can1*.

Oligo name	Sequence (5' to 3')	Purpose
Can1-E541A D543Af	CCACTGCGGTCGCCGGGGTATTTTTTCACCG	SDM forward primer for E541A D543A
Can1-E541A D543Ar	ATACCCCGGCGACCGCAGTGGATTGCGGGG	SDM reverse primer for E541A D543A
Can1-R206Ef	GCCAAAGATATGTCAGAAAAGGCTTTAAGTG	SDM forward primer for R206E
Can1-R206Er	CACTTAAAGCCTTTTCTGACATATCTTTGGC	SDM reverse primer for R206E
Can1-R249Ef	GAATGTATTCGCAGAGTGGCGTGCCTGG	SDM forward primer for R249E
Can1-R249Er	CCAGGCACGCCACTCTGCGAATACATTC	SDM reverse primer for R249E
Can1-K90Ef	CTGGAGGAGCAGAATACTGGGCG	SDM forward primer for K90E
Can1-K90Er	CGCCCAGTATTCTGCTCCTCCAG	SDM reverse primer for K90E
Can1- N12A	GCCTTCTTGGGGCCGATCCTGCC	SDM forward primer for N12A
Can1- N12A-complement	GGCAGGATCGGCCCCAAGAAGGC	SDM reverse primer for N12A
Can1- W42A	CGTTCCCAGCAGCGAACGAGGAGTACG	SDM forward primer for W42A
Can1- W42A- complement	CGTACTCCTCGTTCGCTGCTGGGAACG	SDM reverse primer for W42A
Can1- H113A	AGTTGAAGGAGCTCGTGCCCTTGAGGC	SDM forward primer for H113A
Can1- H113A - complement	GCCTCAAGGGCACGAGCTCCTTCAACT	SDM reverse primer for H113A
Can1- Q222E	CGCTTAGGGGGTGAGTTGTGCTTGCCC	SDM forward primer for Q222E
Can1- Q222E- complement	GGGCAAGCACAACTCACCCCCTAAGCG	SDM reverse primer for Q222E

Supplementary Table 3. Data collection and refinement statistics for Can1 in complex with cA_4 (6SCE).

	Can1 with cA_4
**Data collection**	
Space group	$P\ 2_1\ 2_1\ 2_1$
Cell dimensions	
a, *b*, *c* (Å)	84.4, 84.5, 123.1
α, β, χ (°)	90, 90, 90
Resolution (Å)	59.7-1.83 (1.86-1.83)*
R_{merge}	0.07 (1.43)
$I / \sigma I$	18.0 (2.0)
Completeness (%)	100 (100)
Redundancy	13.3 (13.4)
**Refinement**	
Resolution (Å)	69.7-1.83
No. reflections	74943
R_{work} / R_{free}	0.18 / 0.21
No. atoms	
Protein	4892
Ligand/ion	
Water	414
B-factors	88
Protein	42.5
Ligand/ion	25.4
Water	47.0
R.m.s. deviations	
Bond lengths (Å)	0.010
Bond angles (°)	1.42

*Values in parentheses are for highest-resolution shell.
