# Supplementary material for: Structure and mechanism of a Type III CRISPR defence DNA nuclease activated by cyclic oligoadenylate
Source: Nat Commun. 2020 Jan 24;11:500. doi: 10.1038/s41467-019-14222-x (PMC6981274; doi:10.1038/s41467-019-14222-x)
Supplement: Supplementary file 3 — Reporting Summary [file 41467_2019_14222_MOESM3_ESM.pdf]

nature research

Corresponding author(s): Tracey Gloster
Malcolm White

Last updated by author(s): Jul 24, 2019

Reporting Summary

Nature Research wishes to improve the reproducibility of the work that we publish. This form provides structure for consistency and transparency in reporting. For further information on Nature Research policies, see Authors & Referees and the Editorial Policy Checklist .

Statistics

For all statistical analyses, confirm that the following items are present in the figure legend, table legend, main text, or Methods section.

n/a	Confirmed	
☐	☒	The exact sample size (*n*) for each experimental group/condition, given as a discrete number and unit of measurement
☐	☒	A statement on whether measurements were taken from distinct samples or whether the same sample was measured repeatedly
☒	☐	The statistical test(s) used AND whether they are one- or two-sided<br>*Only common tests should be described solely by name; describe more complex techniques in the Methods section.*
☒	☐	A description of all covariates tested
☒	☐	A description of any assumptions or corrections, such as tests of normality and adjustment for multiple comparisons
☒	☐	A full description of the statistical parameters including central tendency (e.g. means) or other basic estimates (e.g. regression coefficient) AND variation (e.g. standard deviation) or associated estimates of uncertainty (e.g. confidence intervals)
☒	☐	For null hypothesis testing, the test statistic (e.g. *F*, *t*, *r*) with confidence intervals, effect sizes, degrees of freedom and *P* value noted<br>*Give P values as exact values whenever suitable.*
☒	☐	For Bayesian analysis, information on the choice of priors and Markov chain Monte Carlo settings
☒	☐	For hierarchical and complex designs, identification of the appropriate level for tests and full reporting of outcomes
☒	☐	Estimates of effect sizes (e.g. Cohen's *d*, Pearson's *r*), indicating how they were calculated

Our web collection on statistics for biologists contains articles on many of the points above.

Software and code

Policy information about availability of computer code

Data collection	Gels were scanned by Typhoon FLA 7000 imager (GE Healthcare). X-ray data were collected using the standard beamline software (GDA) on beamline I04-1 at Diamond Light Source. SAXS data was collected on beamline B21 at Diamond Light Source, UK using GDA. Size-exclusion chromatography was performed using an Agilent HPLC and controlled by Agilent Chemstation software.
Data analysis	Gel bands were quantified using the Bio-Formats plugin 41 of ImageJ as distributed in the Fiji package and were plotted against time using Kaleidagraph.<br>X-ray data were automatically processed with Xia2 using XDS and XSCALE. The data were phased using AutoSol in Phenix and the initial model was built in AutoBuild. Model refinement was carried out using REFMAC5 in the CCP4 suite and manual manipulation in COOT. The coordinates for cA4 were generated in ChemDraw (Perkin Elmer) and the library was generated using Acedrg, before fitting in COOT. Model quality was monitored using Molprobity.<br>SAXS images were converted to intensities using in-house analysis software DAWN. DAWN produces 1-D SAXS curves that were further analysed using ScÅtter (www.bioisis.net). SAXS modelling was performed with FOXS and CNS version 1.3.

For manuscripts utilizing custom algorithms or software that are central to the research but not yet described in published literature, software must be made available to editors/reviewers. We strongly encourage code deposition in a community repository (e.g. GitHub). See the Nature Research guidelines for submitting code & software for further information.

Data

Policy information about availability of data

All manuscripts must include a data availability statement. This statement should provide the following information, where applicable:

- Accession codes, unique identifiers, or web links for publicly available datasets
- A list of figures that have associated raw data
- A description of any restrictions on data availability

The final protein model and raw X-ray data presented in this study have been deposited in the Protein DataBank with the accession code 6SCE. The SAXS data has

been deposited in bioisis with the accession codes as CAN1AP, CAN1C4. Other data presented in this study are available from the corresponding authors upon reasonable request.

Field-specific reporting

Please select the one below that is the best fit for your research. If you are not sure, read the appropriate sections before making your selection.

☒ Life sciences ☐ Behavioural & social sciences ☐ Ecological, evolutionary & environmental sciences

For a reference copy of the document with all sections, see nature.com/documents/nr-reporting-summary-flat.pdf

Life sciences study design

All studies must disclose on these points even when the disclosure is negative.

Sample size	The biochemical experiments were conducted with 2 biological and 6 technical replicates. For SAXS, apo-state Can1 was analysed using Size-Exclusion Chromatography (SEC), where the analysis was performed twice. For apo-state SAXS curve, a total of 37 independent measurements were used to make the average curve. For the Can1-cA4 experiments, data were measured from 4 concentrations in batch. In batch mode each concentration was measured under flow using a total of 30 exposures and each exposure was assessed for air exposures. Frames free of air shots were averaged and subtracted from an average of 5 independent buffer (SEC-SAXS buffer with ligand) measurements to produce the final SAXS curve.
Data exclusions	No data were excluded from the biochemical or crystallography work presented. For SAXS, data near the beam stop is excluded from further analysis. This region is defined by non-linearity in the Guinier region. For mono-dispersed systems, the SAS curve at low-scattering vectors, q, is linear with respect to ln(I[q]) vs q. Non-ideal behaviour from aggregation or instrumentation scatter causes a smiling or frown in the residuals of the Guinier fit. Data is rejected manually, as shown in the SAXS supplementary PDF file submitted. Since the SAXS curve is a background subtracted curve, data from high-scattering vectors is rejected based on the presence of negative intensities or non-ideal behaviour in the volume-of-correlation plots.
Replication	All attempts at replication were successful.
Randomization	Randomization is not possible for the biochemical and structural work presented.
Blinding	Blinding is not possible for the biochemical and structural work presented.

Reporting for specific materials, systems and methods

We require information from authors about some types of materials, experimental systems and methods used in many studies. Here, indicate whether each material, system or method listed is relevant to your study. If you are not sure if a list item applies to your research, read the appropriate section before selecting a response.

Materials & experimental systems

n/a	Involved in the study	
☒	☐	Antibodies
☒	☐	Eukaryotic cell lines
☒	☐	Palaeontology
☒	☐	Animals and other organisms
☒	☐	Human research participants
☒	☐	Clinical data

Methods

n/a	Involved in the study	
☒	☐	ChIP-seq
☒	☐	Flow cytometry
☒	☐	MRI-based neuroimaging
