# Supplementary material for: Structure and mechanism of a Type III CRISPR defence DNA nuclease activated by cyclic oligoadenylate
Source: Nat Commun. 2020 Jan 24;11:500. doi: 10.1038/s41467-019-14222-x (PMC6981274; doi:10.1038/s41467-019-14222-x)
Supplement: Supplementary file 4 — Description of Additional Supplementary Files [file 41467_2019_14222_MOESM4_ESM.pdf]

Description of Additional Supplementary Files

File Name: Supplementary Data 1
Description: The raw data for the SAXS study.
