# Supplementary material for: Structure and mechanism of a Type III CRISPR defence DNA nuclease activated by cyclic oligoadenylate
Source: Nat Commun. 2020 Jan 24;11:500. doi: 10.1038/s41467-019-14222-x (PMC6981274; doi:10.1038/s41467-019-14222-x)
Supplement: Supplementary file 5 — Supplementary Data 1 [file 41467_2019_14222_MOESM5_ESM.pdf]

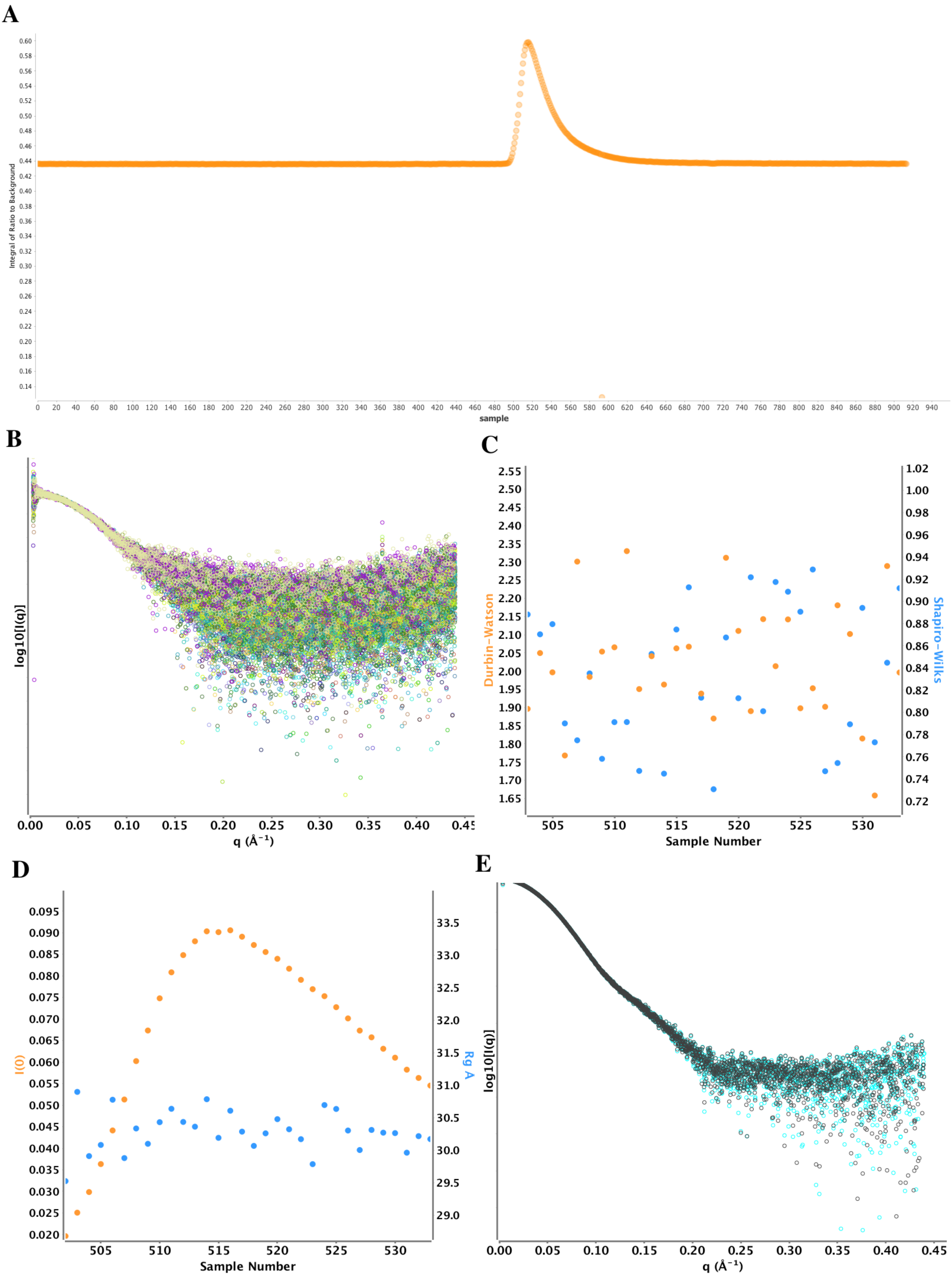

A
Integral of Ratio to Background
sample
B
log10[I(q)]
q (Å⁻¹)
C
Durbin–Watson
Shapiro–Wilks
Sample Number
D
I(0)
Rg A
Sample Number
E
log10[I(q)]
q (Å⁻¹)

Figure | tthb155apo. A. SEC-SAXS Signal Plot. Each point represents the integrated area of the ratio of the sample SAXS curve to the estimated background. **B.** Overlay of SAXS curves of subtracted frames. Each frame is colored based on the following table. **C.** Durbin-Watson and Shapiro-Wilks tests examining the distribution of the residuals between two frames. In this case, comparisons are made in reference to the first frame. Radiation damage or lack of similarity can be observed as a trend in either statistic across the frame set. Likewise, similarity is demonstrated by a random distribution of the statistics. **D.** Double Y plot with I(0), orange, and R_g, cyan, estimated from the Guinier region for each subtracted frame. For a single concentration measurement made over several frames, radiation damage will be observed as an increase in I(0) and R_g. For SEC-SAXS, I(0) should change with the concentration of the particle during elution. **E.** Log*10* intensity plot of subtracted and merged SAXS frames. Black represents averaged buffer frames subtracted from averaged sampled frames. Cyan represents median of the buffer frames subtracted from the averaged sample frames. Poor buffer subtraction leads to a displacement between the two curves at high-*q*

	Guinier Izero	Guinier Rg	
502	1.96E-02 +- 1.2E-04	29.52 +- 0.85	01418_tthb155_KW402p
503	2.51E-02 +- 1.5E-04	30.90 +- 1.37	01419_tthb155_KW402p
504	2.98E-02 +- 1.5E-04	29.91 +- 0.82	01420_tthb155_KW402p
505	3.63E-02 +- 1.5E-04	30.08 +- 0.72	01421_tthb155_KW402p
506	4.41E-02 +- 1.5E-04	30.78 +- 0.64	01422_tthb155_KW402p
507	5.13E-02 +- 1.4E-04	29.88 +- 0.43	01423_tthb155_KW402p
508	6.02E-02 +- 1.6E-04	30.33 +- 0.47	01424_tthb155_KW402p
509	6.73E-02 +- 1.5E-04	30.10 +- 0.36	01425_tthb155_KW402p
510	7.48E-02 +- 1.7E-04	30.43 +- 0.38	01426_tthb155_KW402p
511	8.08E-02 +- 1.6E-04	30.64 +- 0.35	01427_tthb155_KW402p
512	8.48E-02 +- 1.9E-04	30.43 +- 0.43	01428_tthb155_KW402p
513	8.80E-02 +- 1.6E-04	30.36 +- 0.31	01429_tthb155_KW402p
514	9.03E-02 +- 2.0E-04	30.78 +- 0.45	01430_tthb155_KW402p
515	9.01E-02 +- 1.6E-04	30.19 +- 0.25	01431_tthb155_KW402p
516	9.06E-02 +- 1.9E-04	30.61 +- 0.42	01432_tthb155_KW402p
517	8.91E-02 +- 1.8E-04	30.28 +- 0.32	01433_tthb155_KW402p
518	8.72E-02 +- 2.0E-04	30.06 +- 0.44	01434_tthb155_KW402p
519	8.55E-02 +- 2.0E-04	30.26 +- 0.51	01435_tthb155_KW402p
520	8.39E-02 +- 2.3E-04	30.48 +- 0.61	01436_tthb155_KW402p
521	8.17E-02 +- 2.0E-04	30.32 +- 0.47	01437_tthb155_KW402p
522	7.91E-02 +- 1.8E-04	30.17 +- 0.37	01438_tthb155_KW402p
523	7.69E-02 +- 3.1E-04	29.78 +- 1.56	01439_tthb155_KW402p
524	7.53E-02 +- 1.7E-04	30.69 +- 0.41	01440_tthb155_KW402p
525	7.27E-02 +- 1.8E-04	30.63 +- 0.46	01441_tthb155_KW402p
526	7.01E-02 +- 1.8E-04	30.30 +- 0.44	01442_tthb155_KW402p
527	6.73E-02 +- 1.5E-04	30.00 +- 0.38	01443_tthb155_KW402p
528	6.57E-02 +- 1.6E-04	30.31 +- 0.35	01444_tthb155_KW402p
529	6.31E-02 +- 1.8E-04	30.27 +- 0.71	01445_tthb155_KW402p
530	6.10E-02 +- 1.8E-04	30.26 +- 0.54	01446_tthb155_KW402p
531	5.82E-02 +- 1.6E-04	29.96 +- 0.49	01447_tthb155_KW402p
532	5.63E-02 +- 2.0E-04	30.21 +- 0.94	01448_tthb155_KW402p
533	5.45E-02 +- 1.7E-04	30.17 +- 0.50	01449_tthb155_KW402p

apo-Can1

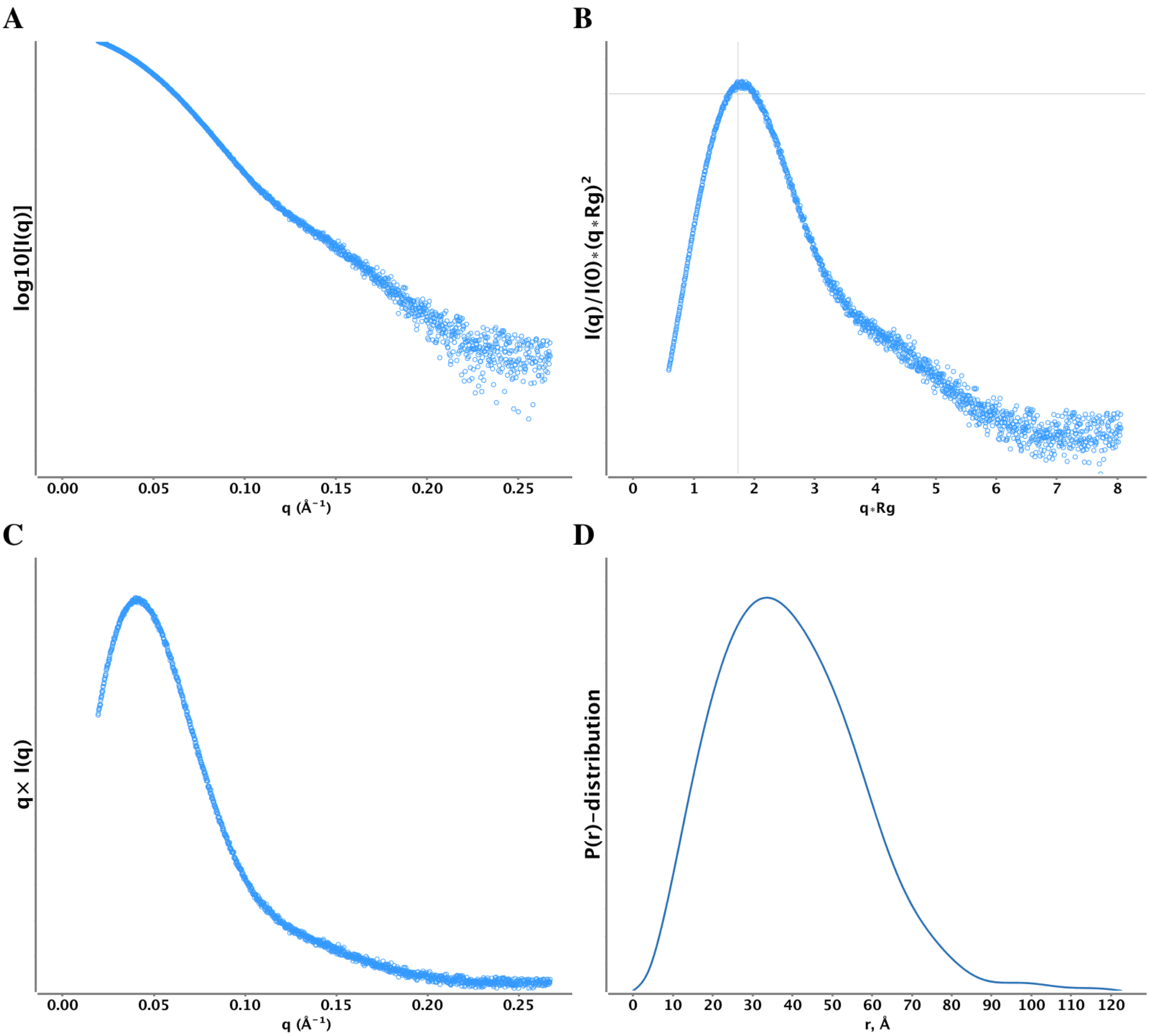

apo-Can1 | Merged peak frames of apo-Can1 (TTHB155) from SEC-SAXS **A.** Log_{10} SAXS intensity versus scattering vector, q. Plotted range represents the positive only data within the specified q-range. **B.** Dimensionless Kratky plot. Cross-hair marks the Guinier-Kratky point (1.732, 1.1), the main peak position for globular particles. **C.** Total scattered intensity plot. Plot readily demonstrates negative intensities at high-q. Over-subtraction of background leads to significant negative intensities. Likewise, under-subtraction can be observed as an elevated baseline at high-q. Horizontal line is drawn at y=0. **D.** Pair-distance, P(r), distribution function. Maximum dimension, d_{max}, is the largest non-negative value that supports a smooth distribution function.

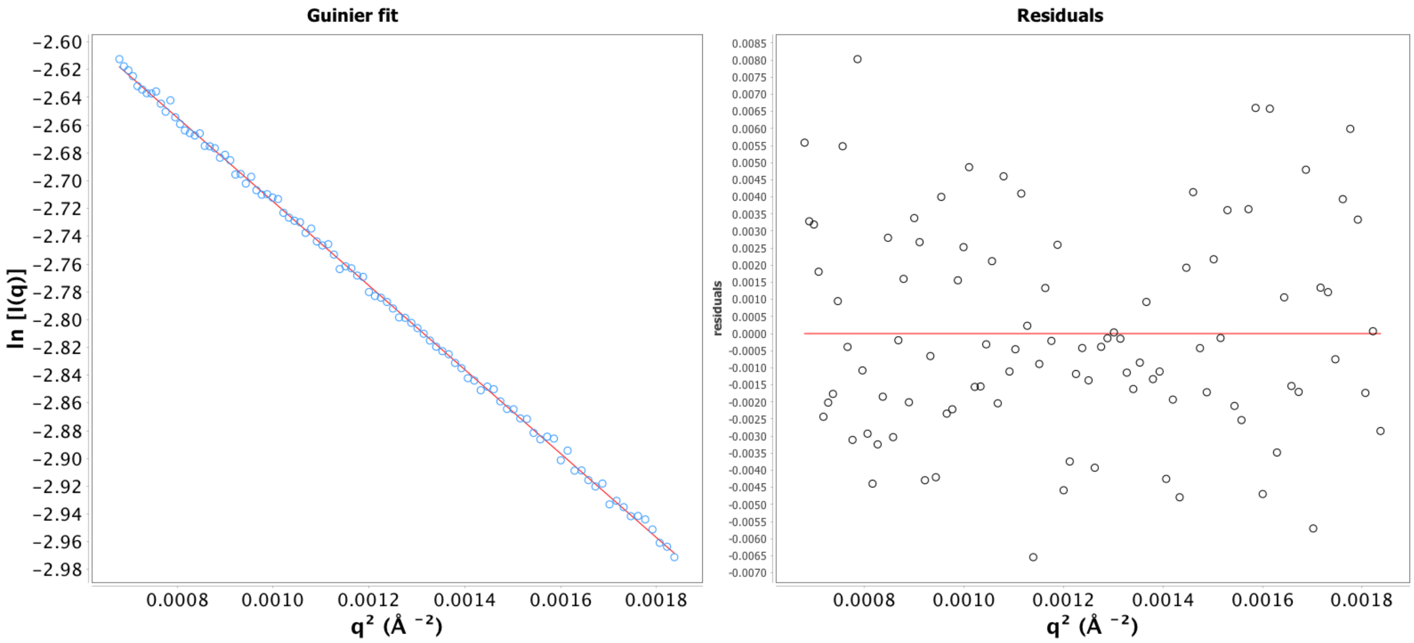

	Reciprocal	Real Space	units
q-min	0.02606720	0.02052910	A^{-1}
q-max	0.04303900	0.26724500	A^{-1}
points(min:max)	95	1352	
Rg	30.13 +- 0.15	30.40 +- 0.00	A
I[zero]	8.96E-02 +- 1.0E-04	8.86E-02 +- 0.0E+00	
Volume	133853	132336	A^3
Vc	508.93	503.16	A^2
	R^2	Chi^2*free*	
Score	1.00	0.94	
Method		Moore L2-NORM	
Background		Yes	
Porod Exponent	3.88 +- 0.04		
d-max	122.5		A
bin-width	11.76		A
Ns	11		
redundancy	130		

All values are reported in non-SI units of Angstroms (A). For biological particles, Porod exponent can only be within 2 and 4 inclusive. Bin-width is the effective, real-space resolution of the P(r)-distribution calculated as d_{max} divided by Shannon number, N*s*.

Bound Can1

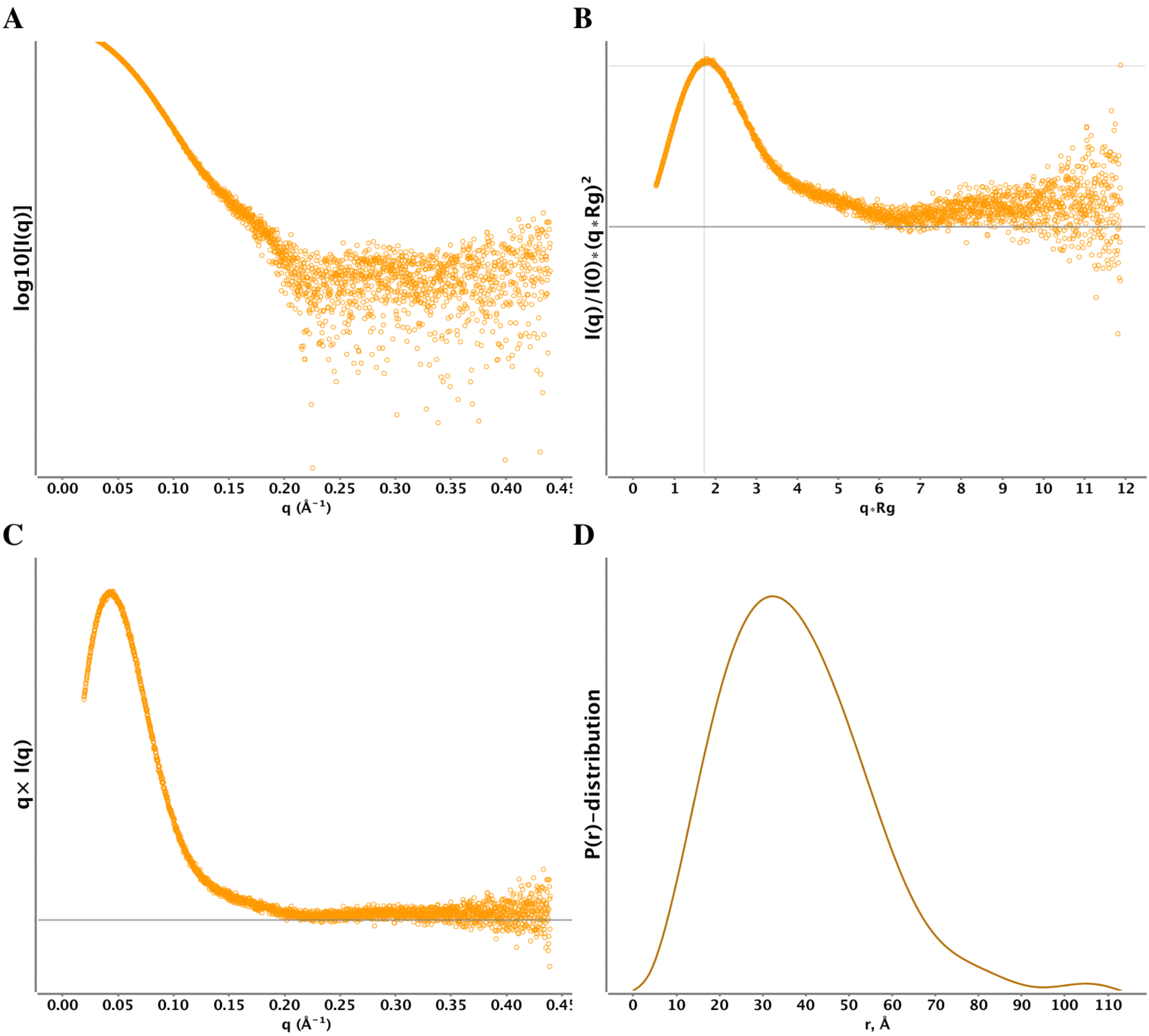

Bound Can1 | SAXS of Can1 with ~3-fold excess of cA4 ligand. Sample was purified by size-exclusion chromatography using Shodex KW-402.5 column at the beamline. Peak fraction was taken and mixed with ligand. Samples were SAXS within 30 minutes of purification. **A.** Log_{10} SAXS intensity versus scattering vector, q. Plotted range represents the positive only data within the specified q-range. **B.** Dimensionless Kratky plot. Cross-hair marks the Guinier-Kratky point (1.732, 1.1), the main peak position for globular particles. **C.** Total scattered intensity plot. Plot readily demonstrates negative intensities at high-q. Over-subtraction of background leads to significant negative intensities. Likewise, under-subtraction can be observed as an elevated baseline at high-q. Horizontal line is drawn at y=0. **D.** Pair-distance, P(r), distribution function. Maximum dimension, d_{max}, is the largest non-negative value that supports a smooth distribution function.

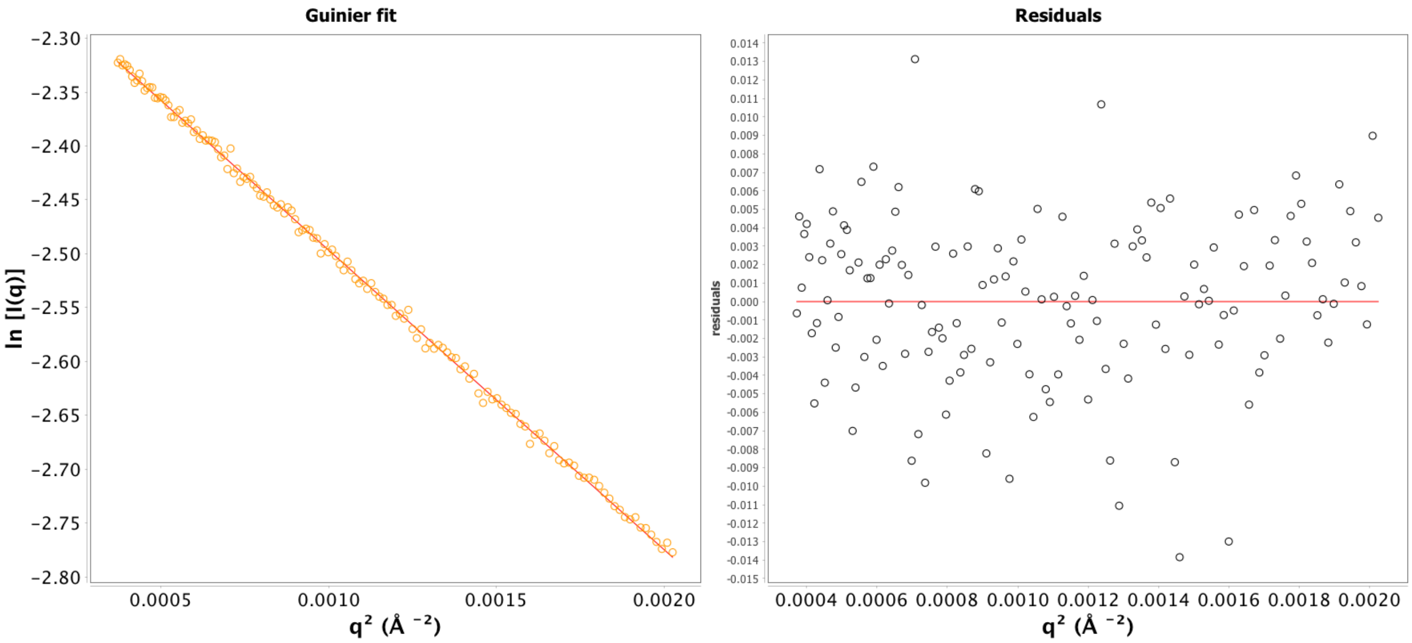

	Reciprocal	Real Space	units
q-min	0.01927853	0.01927853	A^{-1}
q-max	0.04518282	0.25527558	A^{-1}
points(min:max)	145	1322	
Rg	28.88 +- 0.13	29.02 +- 0.00	A
I[zero]	1.09E-01 +- 1.0E-04	1.07E-01 +- 0.0E+00	
Volume	121421	119703	A^3
Vc	489.49	482.56	A^2
	R^2	Chi^2*free*	
Score	1.00	0.75	
Method		Moore L2-NORM	
Background		Yes	
Porod Exponent	3.89 +- 0.04		
d-max	113.0		A
bin-width	12.31		A
Ns	10		
redundancy	144		

All values are reported in non-SI units of Angstroms (A). For biological particles, Porod exponent can only be within 2 and 4 inclusive. Bin-width is the effective, real-space resolution of the P(r)-distribution calculated as d_{max} divided by Shannon number, N*s*.
